# Health Information Technologies—Academic and Commercial Evaluation (HIT-ACE) methodology: description and application to clinical feedback systems

**DOI:** 10.1186/s13012-016-0495-2

**Published:** 2016-09-22

**Authors:** Aaron R. Lyon, Cara C. Lewis, Abigail Melvin, Meredith Boyd, Semret Nicodimos, Freda F. Liu, Nathaniel Jungbluth

**Affiliations:** 1 Department of Psychiatry and Behavioral Sciences, University of Washington School of Medicine, 6200 NE 74th Street, Suite 100, Seattle, WA 98115 USA; 2 Department of Psychological and Brain Sciences, Indiana University, 1101 E. 10th St., Bloomington, IN 47405 USA; 3 Department of Psychology, University of Washington, Guthrie Hall, Box 351525, Seattle, WA 98195 USA

**Keywords:** Health information technology, Measurement feedback systems, Behavioral health, Mental health, Competitive analysis, Routine outcome monitoring

## Abstract

**Background:**

Health information technologies (HIT) have become nearly ubiquitous in the contemporary healthcare landscape, but information about HIT development, functionality, and implementation readiness is frequently siloed. Theory-driven methods of compiling, evaluating, and integrating information from the academic and commercial sectors are necessary to guide stakeholder decision-making surrounding HIT adoption and to develop pragmatic HIT research agendas. This article presents the Health Information Technologies—Academic and Commercial Evaluation (HIT-ACE) methodology, a structured, theory-driven method for compiling and evaluating information from multiple sectors. As an example demonstration of the methodology, we apply HIT-ACE to mental and behavioral health measurement feedback systems (MFS). MFS are a specific class of HIT that support the implementation of routine outcome monitoring, an evidence-based practice.

**Results:**

HIT-ACE is guided by theories and frameworks related to user-centered design and implementation science. The methodology involves four phases: (1) coding academic and commercial materials, (2) developer/purveyor interviews, (3) linking putative implementation mechanisms to hit capabilities, and (4) experimental testing of capabilities and mechanisms. In the current demonstration, phase 1 included a systematic process to identify MFS in mental and behavioral health using academic literature and commercial websites. Using user-centered design, implementation science, and feedback frameworks, the HIT-ACE coding system was developed, piloted, and used to review each identified system for the presence of 38 capabilities and 18 additional characteristics via a consensus coding process. Bibliometic data were also collected to examine the representation of the systems in the scientific literature. As an example, results are presented for the application of HIT-ACE phase 1 to MFS wherein 49 separate MFS were identified, reflecting a diverse array of characteristics and capabilities.

**Conclusions:**

Preliminary findings demonstrate the utility of HIT-ACE to represent the scope and diversity of a given class of HIT beyond what can be identified in the academic literature. Phase 2 data collection is expected to confirm and expand the information presented and phases 3 and 4 will provide more nuanced information about the impact of specific HIT capabilities. In all, HIT-ACE is expected to support adoption decisions and additional HIT development and implementation research.

## Background

Health information technologies (HITs) are rapidly proliferating with respect to their sophistication and capabilities, facilitated, in part, by policies and funding priorities that actively promote or mandate their use and dictate key aspects of their functioning (e.g., [1–3]). Increasingly, HIT innovations are being developed to support the implementation of evidence-based practices across a wide range of healthcare domains (e.g., patient/disease registries, measurement feedback systems, clinical-decision support tools). Unfortunately, there are few frameworks for effectively compiling, evaluating, and integrating information about these technologies, limiting their utility for implementation research and practice.

Existing methodologies for HIT evaluation are few in number, may be too narrow in their application, overemphasize academic—rather than commercial—products and information sources, are inconsistently driven by theory, and/or do not adequately evaluate the mechanisms through which HITs exert their effects (e.g., [4–9]). Models such as the risk assessment framework (RAF) [10], for example, indicate growing interest in HIT synthesis. The RAF addresses the critical issue of the risks posed by specific smartphone apps by articulating different types and level of risk, based on the probability and severity of harm, app complexity, and a variety of contextual factors. However, the RAF is atheoretical and the niche focus of this framework (i.e., patient risk posed by smartphone apps) makes it difficult to apply to HIT more broadly. In another example, the multiphase optimism strategy [11] is a flexible and compelling method for the development of effective and streamlined eHealth interventions based on the performance of potential intervention components across a series of randomized tests over three phases (i.e., screening and selection of components; refining and fine tuning the level of selected components; confirming the efficacy of the final intervention). MOST shows great promise for the development of individual interventions for specific applications, but has less relevance to the generation of generalizable, context-independent knowledge about an entire class of technology which can both inform new technology design or the selection of existing technologies for adoption.

Furthermore, such methodologies—and their associated theories and frameworks—rarely consider all relevant aspects of intended functions, technology design, and implementation; and lack specific processes for evaluating the utility of technologies derived from multiple sectors. Steadily increasing growth in HIT products suggests that development and dissemination largely occurs outside of traditional academic pathways. Indeed, there is evidence from other fields (e.g., solar cells; [12]) that a “commercialization gap” often exists in which commercial technologies rapidly proliferate with little influence from relevant empirical findings. Simultaneously, considerable technological innovation occurs in the commercial sector, but is not incorporated into academic research. New methods of compiling, evaluating, and integrating information from academic and commercial sectors are therefore necessary to provide guidance to developers, researchers, administrators, and other stakeholders who are interested in making informed decisions about system adoption or development of pragmatic HIT research agendas. Finally, existing HIT evaluation studies commonly lack a guiding theoretical framework [13], which limits their coherence, generalizability, alignment with existing literature, and the extent to which core components of HIT and associated mechanisms of implementation can be identified. A theory-informed methodology for compiling, evaluating, and synthesizing HITs from both the academic and commercial sectors would be a significant step forward for the field of implementation science.

### A case example of HIT in need of evaluation and synthesis

Specific subtypes of HITs that support service quality monitoring have been identified among sixteen “quality management strategies” delineated in a recent compilation of evidence-based implementation strategies [14, 15]. Measurement Feedback Systems (MFS [16])—a class of digital HIT that supports the implementation of routine outcome monitoring (ROM) in health service delivery—are one such subtype, and one that is rapidly expanding both in the USA and worldwide, likely due to a growing emphasis on accountability and purposeful uptake by health services agencies [17, 18]. Simultaneously, ROM is increasingly recognized as an evidence-based practice [19–21], and one that may be considered a minimal intervention necessary for change [22]. Consistent with the broad scope of HIT, extant MFS technologies have emerged from disparate sources, serving a panoply of populations and settings, and reflecting diverse academic, service, and commercial interests. The broad scope of these technologies has led to a stark lack of interoperability or consistent standards for use, which presents a significant problem to potential consumers [23]. As a result, information about HIT development, functionality, and implementation readiness is fractured, siloed, and inconsistent, thereby limiting the extent to which available MFS are accessible to stakeholders seeking to implement ROM and inadvertently reducing their ultimate public health impact. No methodologies are available that comparatively evaluate MFS tools. There is a specific critical unmet need to empirically evaluate existing MFS that support ROM—as well as other HIT designed to support the uptake of innovative practices—in order to advance implementation science and practice.

#### Methodology overview and aims

This article presents (1) a theory-informed, structured methodology—Health Information Technologies—Academic and Commercial Evaluation (HIT-ACE)—to support compilation, empirical comparative evaluation, and synthesis of available information for HIT, as well as (2) preliminary results from a case example application to MFS. In brief, this methodology integrates information from the scientific literature, as well as publicly available commercial sources, and draws on existing theories and frameworks to conduct a *competitive analysis*. The competitive analysis method is drawn from marketing and strategic business management [24] to structure and prioritize results. Competitive analysis drives innovation by identifying the strengths and weaknesses of existing products within a particular domain, but has not yet been utilized in implementation science.

The current example application of HIT-ACE is specific to MFS used to support ROM in behavioral health. Consistent with the literature in the USA, where this project was carried out, we use the term “behavioral health” as an overarching term for mental health and substance abuse services [25]. This scope allows for a broad (in that it applies to multiple settings in which MFS have been developed and implemented) yet specific exemplar of the HIT-ACE methodology. The methodology is intended to be generalizable to a broad range of extant HIT (e.g., electronic health records, personal health records, mobile health apps, patient/disease registries, clinical-decision support tools) to advance their theoretical and empirical basis and, ultimately, their capacity to advance implementation science and practice.

## Methods

### Overview of HIT-ACE methodology

**Fig. 1:**
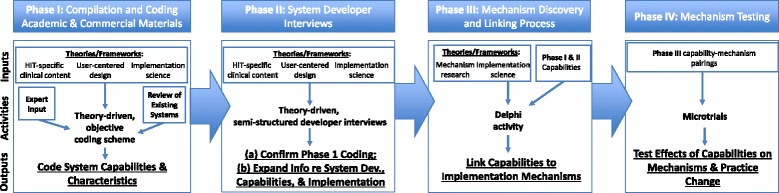
Phases of HIT-ACE methodology

The overall goal of the HIT-ACE methodology is to support the compilation of technologies and their associated capabilities to enable empirical and comparative evaluation and, ultimately, to aid consumers and stakeholders in technology adoption decisions that promote sustainment in service systems. We use the term “consumers” to refer to the individuals who make adoption decisions. In the case of practitioner-facing technologies (e.g., MFS, electronic health records), consumers are likely to be service providers or system administrators. Because HITs are likely to arise from both research and commercial sectors, HIT-ACE is designed to integrate information from both in a single methodological approach with relevance to both the selection of existing technologies or the eventual development of novel technologies. HIT-ACE includes four phases: (1) compilation and coding academic and commercial materials to identify capabilities and characteristics, (2) conducting system developer or purveyor interviews to gather more detailed information about development and implementation processes, (3) a process in which putative implementation mechanisms are linked to HIT capabilities, and (4) experimental testing of HIT capabilities and mechanisms. Earlier phases of the HIT-ACE methodology are designed to be more pragmatic and parsimonious, while later phases address prior phase limitations and are more resource intensive. For instance, phase 2 developer interviews are intended, in part, to evaluate and confirm the findings from phase 1 coding. Figure 1 depicts each phase of the HIT-ACE methodology including inputs, activities, and outputs.

#### Phase 1: coding academic and commercial materials

The first phase of HIT-ACE is focused on developing a theory-driven, objective coding scheme for the capabilities of a given technology. A *capability* is defined as the ability to perform or achieve certain actions or outcomes through a set of controllable and measurable faculties, features, functions, processes, or services. A *characteristic* is defined as distinguishing trait, quality or property. Phase 1 is intended to address descriptive research questions surrounding the nature of a particular type of technology (e.g., How many different products have been developed that may be classified as this type of technology? What are the most common capabilities of the identified technologies?). Although Phase 1 identifies the frequency with which different capabilities and characteristics are present within a given class of technology, no specific weighting is given to more or less commonly occurring aspects. This is because the presence of a capability in a system may be influenced by a multitude of factors (e.g., when and by whom it was designed) and is not necessarily reflective of the importance or effectiveness of that capability. Further, while it is practically useful to gather information about system characteristics (e.g., price, languages available), it is critically important to gather capabilities which are then ultimately linked to putative mechanisms of practice change (phases 3 and 4). The HIT-ACE methodology draws capabilities from multiple sources—including theory, expert opinion, and review of existing systems—with the goal of identifying a broad set of capabilities for subsequent empirical evaluation.

The creation of the coding scheme is integral to Phase 1 of HIT-ACE. To ensure its coherence, generalizability, alignment with existing literature, and potential for aiding the identification of core capabilities of HIT and associated implementation mechanisms, code development should be guided by theory representing at least three levels: (1) theory specific to the intended functions of a given class of HIT, namely its intended mechanisms of influence on user behavior in the service of optimizing health outcomes; (2) general frameworks for developing usable, effective, and compelling technology products; and (3) implementation models, focused on the ways in which a technology and its associated supports can facilitate initial adoption and sustained use within service systems. As an example, we present the theoretical foundations for the HIT-ACE methodology applied to MFS below. However, the frameworks identified are not intended to be prescriptive, as specific technologies may be best evaluated using different models.

#### Phase 2: developer/purveyor interviews

Phase 2 of HIT-ACE is intended to confirm the results of phase 1 coding and gather more detailed information about system development, capabilities, and implementation processes than is often possible from the publicly available sources. Phase 2 also expands on Phase 1 with additional descriptive research questions (e.g., Through what processes—and with what types of stakeholder input—were these technologies developed?). Most consumers are unlikely to move beyond the type of information gathering reflected in phase 1 (albeit in a less systematic way), making phase 2 data especially important to aid stakeholders in making informed adoption decisions. It is likely that developers will have additional information that may contribute to the most accurate depiction of a particular HIT landscape. Phase 2 involves a semi-structured interview—based on identified theories—which gathers specific information about the spread of the identified HIT product. Prior to conducting phase 2 interviews, summaries of phase 1 coding are distributed to respondents for review in order to facilitate clarification and correction of the information collected. Additional interview content in phase 2 aligns with the three domains and frameworks described above (i.e., those specific to the intended functions of the HIT; those focused on design and usability; and those emphasizing successful implementation).

#### Phase 3: linking HIT capabilities to putative implementation mechanisms

In the third phase of the HIT-ACE methodology, specific system capabilities are linked to implementation mechanisms to support future empirical testing (in phase 4). Identification and evaluation of mechanisms of change has become increasingly common in intervention studies [26], but comparable efforts within implementation science are practically non-existent. Mechanisms explain why or how implementation strategies, such as HIT, exert their effects and are critically important for understanding their heterogeneity and contextual dependence [27]. Phase 3 is designed to address exploratory questions surrounding the putative mechanisms through which aspects of a technology affect implementation outcomes (e.g., What mechanisms might be responsible for the anticipated—or previously measured—impact of the technology? Which system capabilities are likely to influence which mechanisms most directly?). Capabilities that are commonly occurring and those present in only a few systems should both be considered during phase 3.

Recent studies have used expert consensus to improve the specificity with which implementation strategies are articulated [15, 28], but very little empirical evidence about the mechanisms through which they operate is available to drive selection or prioritization [29]. For these reasons, phase 3 reflects a *Discovery and Linking Process* with the goal of (a) identifying the mechanisms through which a specific class of HIT exerts its effects on service provider behavior to optimize service recipient outcomes and (b) linking those mechanisms to the capabilities identified in phase 1 and confirmed in phase 2. Candidate mechanisms should be drawn from existing, multilevel frameworks for mechanism and implementation research (e.g., [30–32]) and confirmed via structured input from experts in implementation and the specific class of HIT via a systematic Dephi process [33] to link mechanisms to capabilities (e.g., EMR warnings [capability] to aid in memory recall [mechanism]), and achieve consensus rank-ordered capability-mechanism pairings for phase 4 evaluation.

#### Phase 4: experimental testing of capabilities and mechanisms

Phase 4 is designed to address more causal research questions about the impact of specific capabilities on mechanisms (e.g., does an increase in the intensity or dosage of capability X result in a corresponding increase in mechanism Y?). To accomplish this, Phase 4 involves conducting a series of *microtrials*—rapid and brief tests of the effects of circumscribed environmental or behavioral manipulations on proximal outcomes or mechanisms of change [34]—to evaluate the connection between HIT capabilities and mechanisms identified in phase 3. Recently, microtrials have been used as a feasible method for testing individual parenting techniques (e.g., praise) outside of the context of full treatment packages to determine their discrete merit [35]. Early in a development process, microtrials have the potential to support the collection of “proof of concept” evidence for specific techniques or HIT capabilities using within-subjects, case study designs. Although microtrials represent a feasible approach to engaging in rapid, small-scale prototyping to evaluate components of complex psychosocial processes [36], they have not yet been applied to the analysis of HIT. In HIT-ACE, each microtrial can test the effects of a single, experimentally manipulated capability of the technology on identified mechanisms and provider practice changes expected to result from the technology (e.g., EMR embedded warnings [capability] to remind [mechanism] physician’s about evidence-based prescribing algorithms). It is hypothesized that each capability will have stronger effects when, based on theory, it targets the putative mechanisms of action per phase 3. Overall, phases 3 and 4 aim to utilize and build upon the preliminary findings of phases 1 and 2 while focusing in an increasingly experimental manner on implementation success. In phases 3 and 4, evaluation of which capabilities impact which mechanisms is intended to allow for the identification of a smaller set of core capabilities and, ultimately, more parsimonious and pragmatic technologies.

### Application to MFS: guiding theoretical frameworks

As an example, we provide a description and preliminary results from the application of phase 1 of HIT-ACE to MFS below. We focus only on phase 1 in this paper because a detailed example of all phases would exceed the scope of a single paper and because phase 1 is intended to produce a user-friendly and relatively inexpensive synthesis that addresses the academic and commercial gaps. Phase 2 subsequently addresses phase 1 limitations and expands phase 1 findings, while phases 3 and 4 advance a more extensive, novel research agenda.

The identification of relevant theory in the three major domains described above is critical to phase 1 of the HIT-ACE methodology: (a) intended functions/mechanisms, (b) user-centered design, and (c) implementation science. In the application of HIT-ACE to MFS, we drew from leading feedback theories (i.e., Feedback Intervention Theory [37], Contextualized Feedback Intervention Theory [38]) to inform the review because the provision of feedback is a core MFS function (i.e., the process through which MFS purportedly have an effect). Importantly, in selecting this framework, our research team noted a paucity of potential theoretical frameworks that focused on feedback as a critical component. Among other components, these theories articulate aspects of feedback that are likely to make it maximally effective (e.g., that feedback be provided relative to established standards, given immediately, and include actionable information about how to improve performance).

Second, our approach integrates frameworks and evidence from the growing field of user-centered design. User-centered design is an iterative approach to product development that grounds the process in information about the people and settings that will ultimately use a product [39, 40]. The approach is deeply ingrained in the contemporary discipline of human-computer interaction and the concepts of human-centered design, user experience, and experience design, among others, and is increasingly recognized as an essential component of effective HIT development [41]. Although there are no well-developed theories of user-centered design, there exist a number of frameworks, processes, or compendia of techniques for developing compelling and useful products [42, 43]. Frameworks here were selected for their completeness and breadth with the goal of applicability across systems, given that very little is currently known about design of MFS technologies. Importantly, these frameworks do not necessarily come from any specific health domain, but are intended to be relevant to technology development across sectors.

Third, because the implementation, scalability, and sustainment of technologies is generally a goal of HIT development, the incorporation of implementation science models that articulate (a) aspects of innovations that are likely to enhance their implementability (e.g., relative advantage, acceptability) and (b) outcomes of effective implementation (e.g., adoption, cost-effectiveness) [31, 44, 45] are essential to the review process. Although a large number of implementation frameworks exist [46], they vary in the extent to which they attend to the innovation being implemented. Furthermore, most implementation frameworks attend to a wide variety of constructs at multiple levels of a service system (e.g., inner setting; outer setting [31]). In the current project, implementation frameworks were selected that focused most explicitly on characteristics of the innovation that made it more likely to be adopted and sustained.

### Application to MFS: coding academic and commercial materials (phase 1)

#### Scope

MFS were defined as digital technologies that (1) include, or provide the ability to input into the system, quantitative measures that are administered regularly throughout treatment to collect ongoing information about the process and progress of the intervention, as well as (2) provide an automated presentation of this information and, in doing so, supply timely and clinically useful feedback to mental health providers about their patients and caseloads. As indicated above, we defined the scope of the example application to focus on behavioral health MFS. The behavioral health literature has seen considerable MFS advancement in recent years, including a growing number of MFS publications [47], special issues [48, 49], theories outlining intended use [38], and methods of MFS development [50, 51].

#### MFS compilation/identification

Given the likelihood of information gaps between the commercial and academic sectors, identification of MFS—and of HIT more generally—requires a multi-method process. We engaged in a systematic process to identify MFS in each sector including: (a) Google searches using the search strings below, (b) database searches (i.e., Web of Science, PsycINFO, PubMed), (c) soliciting systems from known experts (i.e., researchers who have published in the area), and (d) email related professional listservs (e.g., Society for Implementation Research Collaboration and the Association of Behavioral and Cognitive Therapies’ Dissemination and Implementation Science Special Interest Group and Technology and Behavior Change Special Interest Group). Search strings used to uncover relevant MFS included “measurement feedback system,” “measurement-based feedback system,” “clinical measurement feedback system,” “outcome monitoring system,” “routine outcome monitoring”, and “system to track outcomes.” Subsequent to MFS identification, all available materials was collected including websites and relevant literature. Identified MFS had to fit the MFS definition described above and had to report facilitating ROM in behavioral healthcare. Identified systems were excluded if it was not possible to locate a website or literature describing the system. If two systems from the same development team had different names or branding but clearly contained the same set of capabilities, the more recent of the two systems was included. In total, as of December 31, 2014, the final list included 49 systems for review.

#### Coding scheme development

Although the HIT-ACE coding scheme—composed of capabilities and characteristics—is primarily deductive in nature, the initial coding scheme was created to evaluate MFS via both inductive and deductive processes [52]. First, system capabilities were extracted from the literature to establish an initial framework. This stage of development incorporated aspects of Feedback Intervention Theory [37], including capabilities related to “feedback timing” (i.e., along what schedule does the MFS provide feedback) and “standard-gap feedback” (i.e., does the MFS provide feedback relative to a norm or standard). In addition, relevant literature on electronic health records and other HIT led to the inclusion of additional characteristics, such as the existence of “patient portals” as well as characteristics such as whether the systems were compliant with the US Health Insurance Portability and Accountability Act (HIPAA) [53–55]. Next, two representative MFS were reviewed to ensure comprehensiveness of the coding system and identify additional characteristics or capabilities. This process led to the inclusion of characteristics related to system marketing (e.g., the availability of a promotional demonstration) and specific data elements tracked within the systems (e.g., service recipient critical events). Finally, stakeholders (e.g., agency administrators either using or desiring to use MFS) and experts (e.g., researchers developing or publishing on MFS) reviewed the list and provided additional characteristics or capabilities to be included.

The final step of coding scheme development required the concrete operationalization of each MFS capability or characteristic in order to support their consistent application, including positive and negative examples. For example, the capability, “Service Recipient Portal to View Outcomes,” was defined as, “A treatment view tailored specifically for service recipient. This must be intended for the recipient, rather than a common portal that can be shared, with a separate login from that of the service provider.” Definitions and examples were refined during the pilot phase of the coding process. Complete tables of the capabilities and characteristics, including definitions, are provided in Tables 1 and 2, respectively. When combined, Tables 1 and 2 represent the complete phase 1 coding scheme.

**Table 1:** List of capabilities

Category	Capability	Definition
Feedback capabilities		
	Outcome monitoring for provider is a prime function	System’s prime function is noted here.
	Immediate feedback timing	System provides immediate feedback (i.e., within seconds; available upon screen refresh) to se to service provider upon data collection as opposed to a couple hours/days later, by mail or email, etc.
	Provides standard gap feedback	Standard-gap feedback provides information to a user that compares data contained within system to information derived from an external source. This included standard gaps to norms, prior expectation, past performance, performance of other groups, ideal goal.
	Alerts to provider	Alerts are made to service provider in order to bring critical information to the user’s attention in ways that circumvent the usual pathway of providing information. May include emails, pop- ups, flags, etc.
	Corrective feedback from system	System provides corrective feedback (i.e., feedback aimed at changing a provider's approach, strategy or treatment decision) to service provider with the aim of producing a more positive treatment outcome.
	Makes referrals	System facilitates referrals for additional services (i.e., those other than the reason why the MFS- facilitated contact occurred) either in-house (within an agency) or to a different organization
	Compares service providers to other providers	System is able to compare users to other providers in various ways e.g., how often providers use system, how compliant they are to system.
	Alerts to others	Alerts are made to individuals other than the service provider, i.e., supervisors, guardians, etc.
	Compares treatment outcomes to user defined goals	System is able to compare treatment outcomes across time to previously established individual treatment targets.
Data capabilities		Capabilities of the MFS related to how data can be displayed, disseminated, and manipulated
	Summary reports	System creates a static snapshot of relevant information, likely designed for (1) paper chart documentation or (2) sharing with some party (e.g., supervisor, insurance company, client). This report will likely include only a subset of the information available in system.
	Displays outcomes as graphs	System has ability to produce a graphic display of various outcomes.
	Aggregate data at multiple levels	System is able to present data on various levels beyond the individual treatment recipient level, e.g., by treatment provider, center, measure, etc.
	View option of treatment recipient	System gives service provider the ability to view a single client’s relevant information.
	Summary reports for service recipient	A static summary report specifically designed to be shared with the service recipient.
Customizability capabilities		Capabilities associated with how and what aspects of the MFS can be altered to fit a site, provider, or service recipient’s unique needs.
	Library of measures to choose from	System provides two or more measures that users can choose to utilize on a case-by-case or program-by- program basis.
	Provider determines frequency of measure administration	Service provider has the ability to determine how often measures are administered by system; frequency is not set by system.
	New tools and measures can be added	New outcome monitoring tools, instruments, or measures are able to be added to system.
	Ability to create idiographic tracking mechanisms	System has ability to create idiographic tracking mechanisms that may be used to measure progress related to the individual treatment targets recorded by system.
	Customizable dashboard	System user is able to customize and determine what information appears on/in system dashboard.
	Provider can add new tools directly	Individual service providers are able to add new outcome monitoring tools themselves rather than other parties, i.e., supervisors or system administrators.
	Ability to customize alerts	System allows for customizable alerts, e.g., timing of alerts, mode of alert delivery, types of alerts, etc.
Tracking capabilities		Capabilities associated with the MFS’s ability to capture outcomes and processes that are relevant to a service recipient’s progression through treatment.
	Tracks standardized outcomes	Outcomes are specified, quantitative treatment targets that may reasonably be believed to result from the intervention. May include mental/behavioral health (e.g., depression, conduct problems, other symptoms), client functioning across domains (e.g., work, school, social, etc.), physical health, etc. Outcomes may include standardized (i.e., norm-referenced) assessment scales or idiographic (i.e., individualized) outcomes.
	Tracks idiographic measures relevant to treatment process	System is able to track idiographic/non-standardized outcomes (e.g., OCD compulsions, tantrums, self-injury incidents).
	Tracks therapeutic processes	System tracks therapeutic processes related to treatment, e.g., therapeutic alliance, engagement/motivation.
	Tracks interventions delivered by providers	System allows for tracking over time of specified treatment protocol or intervention element/subcomponent use (e.g., exposure therapy, mindfulness exercises, etc.).
	Tracks/measures individual treatment targets (goals)	System is able to track and measure the individual treatment targets/goals that were recorded by the system.
	Records treatment goals	System is able to explicitly record defined individual treatment goals for the service recipient.
	Tracks critical events for service recipient	System allows for indicating the occurrence of important/clinically-relevant events (e.g., suicide attempt, fights with significant others) at discrete points in time regardless of whether these have been previously identified for ongoing monitoring.

**Table 2:** List of characteristics

Category	Characteristic	Definition
Technology		
	Reports system as evidence-based	Coding source states that any aspect of system (e.g., measures, entire systems) is evidence-based.
	HIPAA compliant	Coding source explicitly states that system and its components are HIPAA compliant.
	HL7 compliant	Coding source explicitly states that system is HL7 compliant.
	Adaptive measures	Measures included in system and their included questions are adaptive based on service recipient’s responses.
	Generate invoices for the purposes of billing	System generates invoices based on information within itself.
	System is an EHR	System explicitly states that it is an electronic health record (EHR).
	Reports fulfilling “Meaningful Use” criteria	Coding source explicitly states that system fulfills “Meaningful Use” criteria.
	Reports system as Blue Button Compliant	Coding source explicitly states that system is Blue Button Compliant.
	Dashboard view option	A dashboard is a single-screen display of the most critical information about a provider’s caseload, updated regularly or in “real time.”
	Messaging system for treatment providers	System provides a built-in messaging system for users, e.g. instant messaging, email, etc.
	Integration with other technologies	System has ability to be integrated/used with other similar technologies, including electronic health records.
Training and technical support		
	Available training for system use other than demo	System or creating organization provides additional training related to the use of system capabilities and/or the integration of system into agency or organizational workflows. This training occurs one-time and may include remote (e.g., webinar-based) or in-person training.
	Available technology support	Tech support involves the availability of individuals with extensive experience in the navigation/use of system itself and problem solving related to issues with the technology of itself.
	Available instruction manual for system	There is an available and freely accessible instruction manual for system.
	Ongoing support beyond technical support	System or its creating organization provides ongoing support for the implementation of system and its integration into provider workflows, organizational policies, etc. (e.g., continued consultation about its use in clinical care, administrator decision-making based on aggregated data). This support is ongoing over time.
Administration and use options		
	Internet-based	System is fully web-based, accessible via a browser, and is updated without requiring a download to a local machine or device.
	Free standing software	System is software that “lives” on a local machine/device (e.g. Microsoft Word) that must be updated by user.
	Ability to use on different devices	System has ability to be used on multiple devices/platforms.
	Ability to use on mobile devices	System has ability to be used on mobile devices, e.g. PDA, phone, tablet, etc.
	Available service recipient portal for data entry	Service recipients are able to enter data directly into system via a dedicated portal (e.g. log-in in waiting room to complete measures before therapy session).
	Permission-based log-in for different users	System allows users to provide information remotely through password-protected logins, e.g. service recipient, clinic director, family members, etc.
	Available paper format	System facilitates the completion of measures by service recipients via paper and pencil rather than with a computer or mobile device.
System acquisition		
	Available for purchase/acquisition	System is currently available for purchase or acquisition.
	Available demo of system for promotional purposes	A demo of system is available without requiring purchase or acquisition of system.
	Contact information of developer	Coding source provides contact information for system’s developer.
Accessibility		
	Available in other languages	System has built-in, automatic availability in at least 1 language other than English.
	Provisions for disabled populations	System contains built-in, automatic capabilities to support its accessibility to disabled populations without the need for additional assistive devices (e.g., visually impaired).

#### Pilot and revision

Prior to the formal coding process, the coding system was piloted to test its feasibility and accuracy and to drive refinements. This process was completed by six of the seven authors (A.R.L., C.C.L., M.B., A.M., F.F.L., and N.J.), who independently applied the coding scheme with representative systems and the primary MFS information source (typically a commercial website). After completion of pilot coding for one system, coders met to compare codes, discuss questions, identify possible new codes, and resolve conflicting ratings. Next, revisions were made to the coding scheme to accommodate additional relevant capabilities and to consolidate similar or redundant capabilities. For example, the capability “Alerts to Providers,” defined as “critical information brought to the attention of the mental health provider in a way that differs from how information is usually presented in the system (e.g., flags/highlighting, emails, pop-ups, etc.),” was complemented with a new code, “Alerts to Others,” in order to capture system alerts directed to other recipients (e.g., supervisors).

#### Coding process

As the next component of phase 1, the coding scheme was applied to evaluate identified technologies. In the case of these MFS, all systems were coded by two independent research assistants using the same information sources, including system websites and empirical articles (see Table 3 for a full list of systems and primary coded source). As needed, the two raters consulted with the investigative team to resolve discrepancies. To facilitate coding feasibility, efficiency, and consistency, a decision was made by the investigative team to code the most information-rich source in an attempt to maximize accuracy in the information accessed. The primary information source was then reviewed in full, including all videos, links, and appendices where relevant, by two independent raters.

**Table 3:** Representative publications and information sources

System	Publications/web resources
ACORN	http://psychoutcomes.org/
AKQUASI	https://www.klinikum.uni-heidelberg.de/index.php?id=137798
ALERT	Brown, G.S., Lambert, M.J., Jones, E.R., and Minami, T. (2005). Identifying highly effective psychotherapists in a managed care environment. The American Journal of Managed Care, 11(8), 513–520.
Assessment Center	https://www.assessmentcenter.net/
BASIS-24	http://ebasis.org/basis24.php
Behavior Monitoring Assessment System (BIMAS)	http://www.mhs.com/product.aspx?gr=edu&id=overview&prod=bimas
Brief Problem Monitor (BPM)	http://www.aseba.org/bpm.html
Care Management Tracking System (CMTS)	http://aims.uw.edu/resource-library/care-management-tracking-system-cmts
Carepaths	http://blog.carepaths.com/
CelestHealth System	Bryan, C.J., Kopta, S.M., & Loews, B.D. (2012). The CelestHealth System. Integrating Science and Practice, 2(2), 8–11.
Centervention	https://centervention.org
Child Health and Development Interactive System (CHADIS)	http://www.chadis.com/index.html
Collaborative Mental Health Management Enhanced Dashboard (COMMEND)	Lindley, S.E. and Wang, D.Y. “COMMEND: Collaborative Mental Health Management Enhanced Dashboard.” Presentation.
Computer-based Health Evaluation System (CHES)	http://ches.at/ches/index.php
Contextualized Feedback Systems (CFS)	http://peabody.vanderbilt.edu/research/center-evaluation-program-improvement-cepi/contextualized_feedback_systems_cfs.php
CORE Outcome Measure (CORE-OM)	http://www.coreims.co.uk
CROMIS	http://ca.linkedin.com/pub/david-ross/10/601/b0b
Clinical Dashboard	Chorpita, B.F., Bernstein, A., Daleiden, E.L., and The Research Network on Youth Mental Health. (2008). Driving with roadmaps and dashboards: Using information resources to structure the decision models in service organizations. Administration and Poliy in Mental Health and Mental Health Services Research, 35, 114–123.
DIALOG	Priebe, S., McCabe, R., Bullenkamp, J., Hansson, L., Lauber, C., Martinez-Leal, R., et al. (2007). Structured patient-clinician communication and 1-year outcome in community mental healthcare: Cluster randomized, controlled trial. The British Journal of Psychiatry, 191, 420–426.
Evidence-Based Assessment System for Clinicians (EAS-C)	Smith, R.E., Fagan, C., Wilson, N.L., Chen, J., Corona, M., Nguyen, H., Racz, S., and Shoda, Y. (2011). Internet-based approaches to collaborative therapeutic assessment: New opportunities for professional psychologists. Professional Psychological Research and Practice, 42(6), 494–504.
Functional Assessment Systems (FAS)	http://www.fasoutcomes.com/
Innerlife	http://www.innerlife.com/index.asp
Intra/Compass	Lueger, R.J. (2012). The Integra/COMPASS Tracking Assessment System. Integrating Science and Practice, 2(2), 20–23.
MHITS	Unützer, J., Choi, Y., Cook, I.A., and Oishi, S. (2002). Clinical computing: A web-based data management system to improve care for depression in a multi-center clinical trial. Psychiatric Services, 53(6), 671–678. School-Based Mental Health Integrated Tracking System (SB-MHITS). Unützer, J. GA-U Mental health pilot: Integrating primary care and mental health.
Mobile Therapy	http://www.mobiletherapy.com/
My Outcomes	http://www.myoutcomes.com/
OQ Measures	http://www.oqmeasures.com/
Outcome Tracker	www.outcometracker.org
Owl Outcomes	http://owloutcomes.com
Partners for Change Outcome Management System (PCOMS)	https://heartandsoulofchange.com/
Penelope	http://www.athenasoftware.net
Polaris-BH	http://www.polarishealth.com/products/behavioral-health/
Polaris-CD	http://www.polarishealth.com/polaris-cd-chemical-dependency/
PQRS PRO	http://apapo.pqrspro.com/APAPOGS
PracticeWise	http://www.practicewise.com/
Psychological Outcome Profiles (PSYCHLOPS)	http://www.psychlops.org.uk
SumOne for Kids	Beck, S.A., Meadowcroft, P., Mason, M., and Kiely, E.S. (1998). Multiagency outcome evaluation of children's services: A case study. The Journal of Behavioral Health Services and Research, 25(2), 163–176.
Systemic Therapy Inventory of Change (STIC)	Pinsof, W.M., Zinbarg, R.E., Lebow, J.L., Knoblock-Fedders, L.M., Durbin, E., Chambers, A., et al. (2009). Laying the foundation for progress research in family, couple, and individaul therapy: The development and psychometric features of the initial systemic therapy inventory of change. Psychotherapy Research, 19(2), 143–156.
Telesage Outcomes Measurement System	http://web.telesage.com/mental-health-outcomes.php
Texas Children’s Mental Health Plan (TCMHP)	Rouse, L.W., Toprac, M.G., and MacCabe, N.A. (1998). The development of a statewide continuous evaluation system for the Texas Children's Mental Health Plan: A total quality management approach. The Journal of Behavioral Health Services and Research, 25(2), 194–207.
The Schwartz Outcome Monitoring	Blais, M.A. (2012). The Schwartz Outcome Scale-10 (SOS)-10. Integrating Science and Practice, 2(2), 40–42. Overington, L. and Ionita, G. (2012). Progress monitoring measures: A brief guide. Canadian Psychology, 53(2), 82–92.
Therapy Rewind	https://www.therapyrewind.com
Tool Kit	https://www.ebptoolkit.com/
Treatment Outcome Package	Youn, S.J., Kraus, D.R., and Castonguay, L.G. (2012). The Treatment Outcome Package: Facilitating practice and clinically relevant research. Psychotherapy, 49(2), 115–122. Kraus, D.R., Seligma, D.A., and Jordan, J.R. (2005). Validation of a behavioral health treatment outcome and assessment tool designed for naturalistic settings: The Treatment Outcome Package. Journal of Clinical Psychology, 61(3), 285–314. Treatment Outcome Package (TOP): Treatment Outcome Package – Substance Abuse (TOP-SA) Fact Sheet.
Treatment Progress Indicator (TPI)	Tuso, P. (2014). Treatment Progress Indicator: Application of a new assessment tool to objectively monitor the therapeutic progress of patients with depression, anxiety, or behavioral health impairment. The Permanente Journal, 18(3), 55–59.
Treatment Response Assessment for Children (TRAC)	http://www.albertafamilywellness.org/system/files/report-files/john_weisz_ppt_for_ebbd_symposium.pdf
Valant	http://valant.com/
VitalHealth	http://www.vitalhealthsoftware.com/products/questlink
Wrap Around Team Monitoring	http://depts.washington.edu/wrapeval/WFI.html

*Note:* The information in this table represents the coding material for each system that was used in phase 1.

**Fig. 2:**
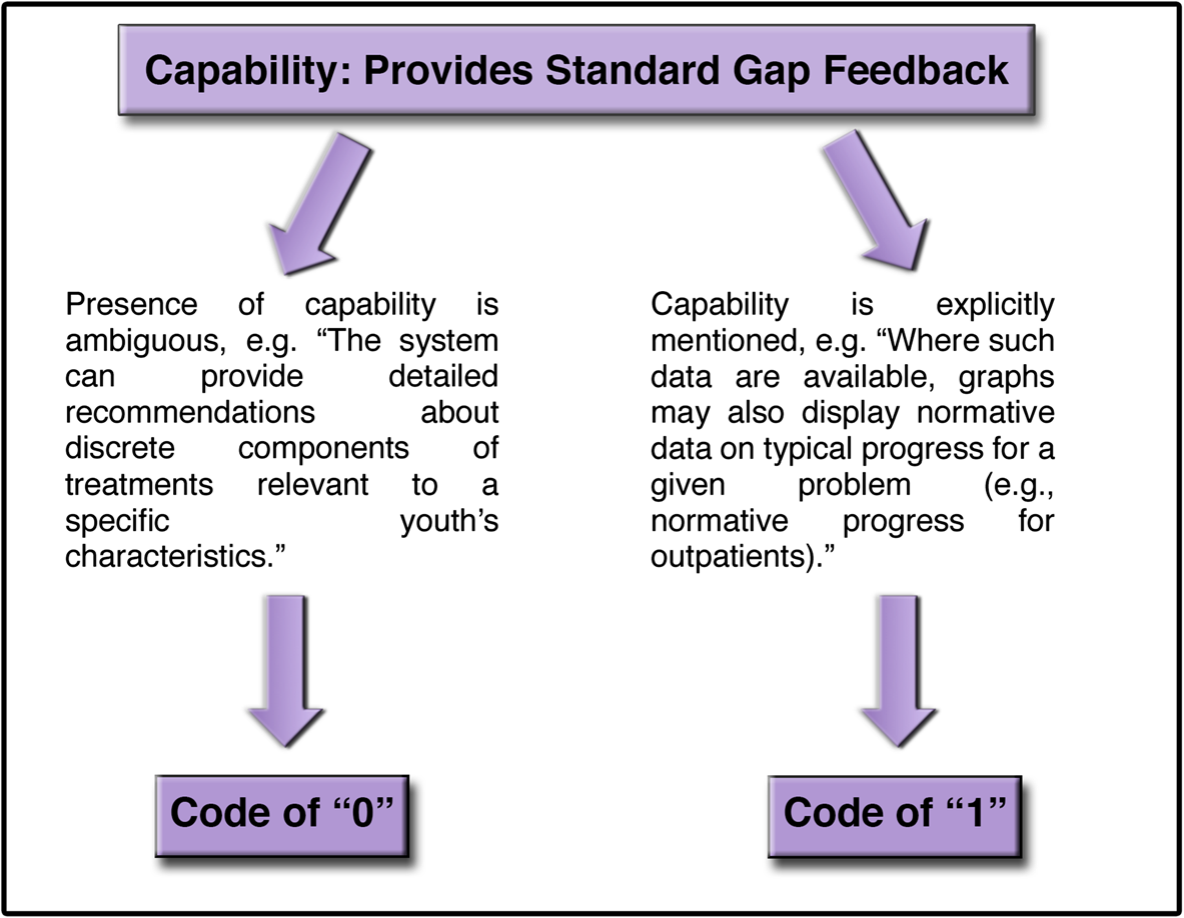
Coding process example

**Fig. 3:**
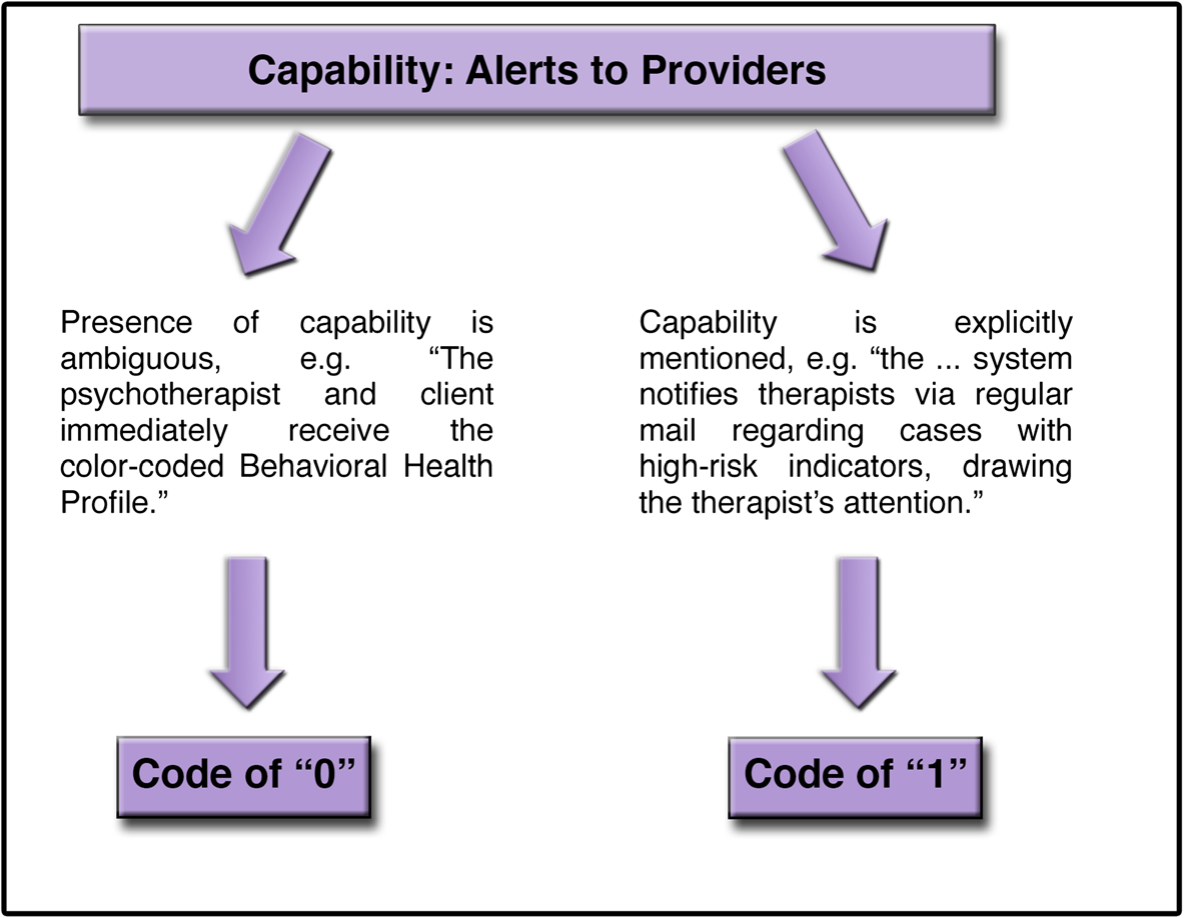
Coding process example

A consensus coding process was used in which raters reviewed materials independently and then met to arrive at consensus judgments through open dialogue [56, 57]. All capabilities and characteristics were assigned a “0” or a “1” to reflect its absence or presence, respectively. Coding was intended to reflect the viewpoint of a potential consumer in that, if the presence of a capability was unclear or not mentioned, a consumer would likely assume that the system lacked this capability. Therefore, a “0” was given if the information about a capability/characteristic was too ambiguous, the capability was not mentioned at all, or if it was stated explicitly that the system did not have this capability (although few systems explicitly mentioned not having a particular capability). A “1” was given if the MFS clearly had the capability. See Figs. 2 and 3 for a visual representation of this process. The dichotomous ratings for each of the capabilities were analyzed to determine the frequency with which various capabilities were represented across the systems. In addition to dichotomous coding, additional qualitative information was collected to further describe the capabilities and characteristics identified as present or absent. For example, specific information was collected about the assessment measures contained within each MFS, types of standard-gap feedback provided, and the extent to which provider interventions/practices were tracked.

#### Bibliometric data

We also examined the number of published articles referring to each system. Relevant articles were obtained by searching the full system name—and acronym if applicable—in quotation marks in Web of Science and PsycInfo. Articles were included if the research described, used, developed, tested or reviewed the system, but not included if the article only cited or made reference to the existence of a system. This search method was intended to provide a snapshot of the frequency with which the system was discussed in the literature, but not necessarily an indicator of the scientific rigor of the system. Additionally, locating these articles made it possible to systematically track the emergence of a subset of these systems over time. Exact data related to the inception of these systems were difficult to locate due to the fact that public information often does not include creation or development history. However, all relevant articles for each system were reviewed through the process described above and the earliest publication date was treated as a proxy for the “inception date,” as it is likely that the publication date of a relevant article is correlated with actual system development.

## Results and discussion

Using phase 1 of the HIT-ACE methodology, a total of 49 systems were identified through the systematic review of product websites and scientific literature, as well as solicitation from experts and professional listservs. Although HIT-ACE does not make assumptions about the importance or effectiveness of capabilities or characteristics based on their frequency of occurrence, the application of phase 1 provides a detailed account of the contemporary MFS landscape for subsequent evaluation. The consensus-coded data for all systems revealed substantial variability in system capabilities, with only 12 of the 56 capabilities and characteristics present in at least half of the systems (Table 4). One explanation for the relative low frequency of some capabilities and low overlap across systems is that the coded capabilities were drawn from multiple literatures. Knowledge of these literatures may have varied based on the professional backgrounds of the individuals involved in any given development team. Publication and dissemination of our findings could help spur and focus development of capabilities currently lacking in the majority of MFS.

**Table 4:** Capability and characteristic frequency

Capability/characteristic	Number of systems with capability/characteristic	Percentage of systems with capability/characteristic
Tracks standardized outcomes	46	93.88
Outcome monitoring for provider is a prime function	45	91.84
Contact info of developer	43	87.76
Internet-based	41	83.67
Reports system as evidence-based	41	83.67
Library of measures to choose from	35	71.43
Summary reports	34	69.39
Displays outcomes as graphs	34	69.39
Aggregate data at multiple levels	30	61.22
View option of treatment recipient	28	57.14
Immediate feedback timing	27	55.10
Available for purchase/acquisition	27	55.10
Provides standard gap feedback	22	44.90
Available training for system use other than demo	22	44.90
Available tech support	22	44.90
Alerts to provider	21	42.86
Available demo of system for promotional purposes	20	40.82
Ability to use on mobile devices	20	40.82
Available paper format	20	40.82
Available service recipient portal for data entry	18	36.73
Available instruction manual for system	18	36.73
HIPAA compliant	17	34.69
Available in other languages	16	32.65
Permission-based logins for different users	16	32.65
Ability to use on different devices	16	32.65
Dashboard view option	15	30.61
Tracks idiographic measures relevant to treatment process	14	28.57
Integration with other technologies	14	28.57
Tracks therapeutic processes	13	26.53
Corrective feedback from system	13	26.53
Tracks interventions delivered by providers	12	24.49
Summary reports for service recipient	12	24.49
Ongoing support beyond tech support	12	24.49
Tracks/Measures individual treatment targets (goals)	11	22.45
Software-based	11	22.45
Provider determines frequency of measure administration	10	20.41
New tools and measures can be added	10	20.41
Records treatment goals	9	18.37
Makes referrals	9	18.37
Adaptive measures	8	16.33
Compares service providers to other providers	7	14.29
Ability to create idiographic tracking mechanisms	7	14.29
Alerts to others	5	10.20
Customizable dashboard	5	10.20
Messaging system for treatment providers	5	10.20
HL7 compliant	5	10.20
Generates invoices for billing purposes	5	10.20
Provider can add new tools directly	4	8.16
Available service recipient portal to view outcomes	4	8.16
Tracks critical events for service recipient	3	6.12
Compares treatment outcomes to user-defined goals	3	6.12
Ability to customize alerts	3	6.12
Reports fulfilling “Meaningful use” criteria	3	6.12
Accessible to disabled populations	2	4.08
System is an electronic health record	2	4.08
Blue Button Compliant	0	0.00

However, another explanation is that the wide range of system capabilities and characteristics observed reflects the diversity of needs that often exist across populations and settings and the range of solutions designed to meet those needs within MFS technologies. For example, the two MFS that also function as electronic health records offered fewest additional capabilities (average per EHR MFS = 6.50 versus average per MFS = 9.77). Additionally, MFS that are not available for purchase or acquisition and were therefore likely created solely for the needs of a specific (research) project, possessed fewer capabilities than MFS that are publically available (average per MFS not publically available = 16.27 versus average per MFS publically available = 20.63).

It is also possible this variability in capability representation across MFS is due to the fact that little is known about which capabilities are core/central and which are auxiliary given the dearth of literature focused on mechanisms of MFS. It is unsurprising that “Tracks Standardized Outcomes” and “Outcome Monitoring to Provider is the Prime Function” were the top two capabilities being represented in 93.9 and 91.8 %, respectively, given that these are the defining features of an MFS. However, the next most common capabilities were “Library of Measures to Choose From” (represented in 71.4 % of MFS), followed by provision of “Summary Reports” and “Displays Outcomes as Graphs” (both represented in 67.4 % of MFS). MFS-relevant theory (e.g., FIT, CFIT) would not support this pattern of most common capabilities. Rather, if guided by FIT [34], we might except to see “Compares Treatment Outcomes to User-Defined Goals,” “Corrective Feedback from System,” and Immediate Feedback Timing” as common among MFS. Subsequent phases of HIT-ACE (phases 3 and 4) are designed to address this gap and determine which capabilities drive system performance in terms of improving the implementation of health innovations and patient outcomes. Therefore, at this time, it is premature to conjecture whether the current array of MFS is fit for purpose.

Finally, because capabilities and characteristics were only coded as present if they were explicitly mentioned in the materials reviewed, it is possible that systems actually contain more capabilities and characteristics than were captured and that there is more overlap in capabilities among existing MFS than documented in the current findings from phase 1. It is for this reason that phase 2 is designed to compare and confirm our preliminary phase 1 coding in addition to gathering more detailed information about the systems. However, it is unlikely that validating the phase 1 results would reveal equivalence in MFS capability representation.

Beyond capability presence, our coding revealed the number of relevant academic articles for each system, which ranged from 0 to 231 (median = 1.5; mode = 0) with 31 systems appearing in the literature and 18 systems having no associated published literature. Three or more relevant articles could be located for 25 systems (51 %). This wide range of published articles for each system reflects the considerable variability in the empirical foundation for available MFS, perhaps indicating the disparate goals (i.e., commercial, academic, etc.) that led to the development of each system. Furthermore, despite high representation for some systems the median and modal values suggest that the “average” system has received relatively little attention from the academic community. These findings highlight the importance of reviewing sources of information beyond the academic literature (e.g., websites, promotional materials) to adequately represent the scope of any specific class or type of HIT. These results also suggest that more research is necessary to evaluate the utility of most of the systems identified. Interestingly, although 31 systems were identified in the scientific literature— and far fewer of which were empirically tested to assess system impact—41 MFS described their system as “evidence based.” Clearly, while there is mounting evidence for the effectiveness of using MFS to support ROM in general, individual systems are likely to vary regarding (a) the scientific rigor with which they were developed and (b) the availability of data regarding their impact on patient and provider behavior or actual clinical outcomes.

**Fig. 4:**
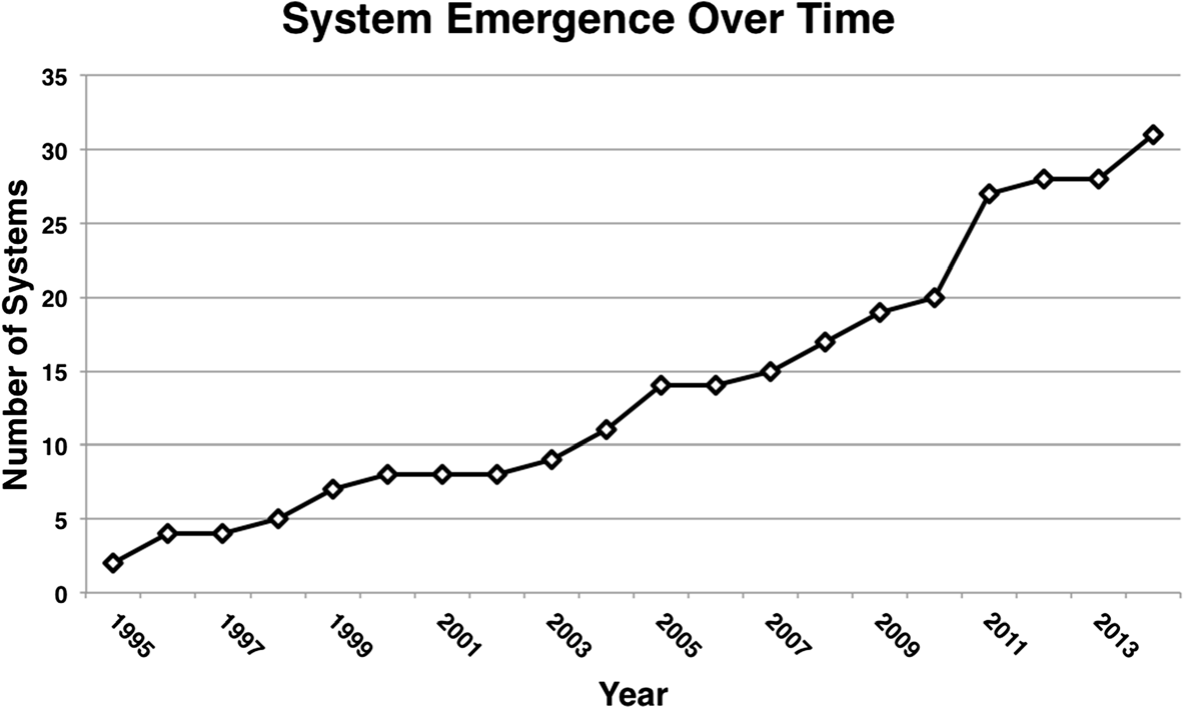
System emergence over time. A *graph* displaying the chronological emergence of systems that were represented in the literature

The published articles also made it possible to approximate the emergence of MFS technologies over time; however, it is important to note that this was only possible for the subset of MFS that were represented in the academic literature (see Fig. 4). Of the 31 systems referenced, the first appeared in 1995. Inception was fairly steady since then, with an average of 1.55 systems emerging in the academic literature every year and relatively linear growth. This apparent steady growth and current volume of MFS technologies demonstrates the need for the development of review methodologies like HIT-ACE, as well as the need for ongoing systematic reviews.

## Conclusions

Given the steady proliferation of HIT—emerging from disparate sources and reflecting diverse academic, service, and commercial interests—a theory-informed, structured methodology to support collation, identification and empirical comparative evaluation for specific classes of HIT would benefit implementation science, health service administrators, and the HIT marketplace. HIT-ACE is one such methodology that can be employed to conduct comprehensive reviews and competitive analyses of any given class of HIT. We described the four phases of the HIT-ACE approach and an example application of phase 1 to available MFS technologies. Preliminary findings clearly demonstrate the utility of HIT-ACE to depict the scope and diversity of MFS beyond what can be identified through a traditional review of the academic literature. Our coding process also revealed substantial vagueness and inconsistency among the publicly available information, highlighting the importance of HIT-ACE phase 2 developer interviews to compare and confirm phase 1 coding and gather additional in-depth information about existing MFS. The variability of capability representativeness from phase 1 (and likely phase 2) highlights the importance of phases 3 and 4, which are designed to facilitate a nuanced—and ultimately experimental—understanding of the mechanisms through which classes of HIT support implementation and impact user behavior and subsequent patient outcomes, reflecting a critical gap in the research and development landscape. This methodology is expected to spur innovation by promoting transparency (e.g., presenting all MFS capabilities currently available on market) in a development space that is historically siloed and fractured.

HIT-ACE is not without limitations. It is a meticulous and labor intensive process that requires significant time and resources to execute, especially for any class of HIT with substantial history, spread, and/or diversity of goals and features. Although the methodology intentionally aims to precede more resource-intensive and potentially expensive methods (e.g., phases 2–4) with those that are less resource-intensive and more feasible (phase 1), it is acknowledged that these methods may be impractical in some contexts, particularly those occurring outside the research setting. Furthermore, by the time all phases of HIT-ACE have been completed, it is possible (if not likely) that new examples of the target technology may have emerged. HIT-ACE is intended to provide a “snapshot” of a given class of technologies at a particular moment in time and to synthesize the available information. Although the HIT landscape tends to evolve rapidly, a detailed “snapshot” is nonetheless valuable given the lack of alternative methodology to gain a comprehensive understanding of the existing marketplace for any given class of HIT.

It is important to note that the nature of the identification process likely did not yield a comprehensive listing of all MFS. However, due to the fact that Google is the most commonly utilized search engine [58] and has been found to produce better results than alternative commercial search engines [59], we believe that it is representative of what a potential consumer or stakeholder may utilize to find systems. Additionally, the English-based databases and search terms utilized—and Google algorithms that prioritize the return of local information [60]—likely resulted in an over-representation of systems based in the USA and potentially excluded other systems originating internationally. Consequently, there is potential for the Phase 1 findings presented herein to be biased toward a US perspective. Nevertheless, we maintain that the HIT-ACE methodology is a potentially powerful tool for systematically evaluating any class of HIT for a variety of research and development purposes as well as health services implementation and dissemination efforts. Related to the latter, HIT-ACE could support the development of rich, regularly updated databases for a given class of HIT that would greatly benefit users trying to select a HIT from among the array of options available or researchers interested in improving technologies through a detailed understanding of their mechanisms of influence.

### Future directions

The results presented herein pertain to HIT-ACE *Phase 1: Coding Academic and Commercial Materials*. Detailed mixed methods evaluation of MFS capabilities and implementation is currently underway in *Phase 2: Developer/Purveyor Interviews*. As a component of this we will be able to link information about MFS to measurable dependent variables related to implementation outcomes (e.g., system penetration into the marketplace, views from consumers and/or stakeholders about system acceptability, feasibility, or appropriateness, etc.). Subsequent to this, we intend to engage in *Phase 3: Linking Putative Implementation Mechanisms to HIT Capabilities*, and *Phase 4: Experimental Testing of Capabilities and Mechanisms*. Phases 3 and 4 of HIT-ACE applied to MFS will be the first attempt, to our knowledge, to isolate core components of HIT, map them to associated mechanisms, and conduct systematic evaluations. These phases will be critical to determine which capabilities maximize MFS outcomes and should be the focus of future development activities. We also envision the development of a living MFS review repository where consumers and developers of all stripes could search for system capabilities and characteristics based on their settings and interests to support adoption-related decision-making and further innovation.

## Abbreviations

HITs: Health information technologies
HIT-ACE: Health Information Technologies—Academic and Commercial Evaluation
MFS: Measurement feedback system(s)
RA: Research assistant
ROM: Routine outcome monitoring

## References

[1] Dimitropoulos L. Health IT research priorities to support the health care delivery system of the future. Rockv MD Agency Healthc Res Qual. 2014. https://healthit.ahrq.gov/sites/default/files/docs/citation/health-it-research-priorities-to-support-health-care-delivery-system-of-future.pdf.

[2] Patient Protection and Affordable Care Act. 2010

[3] Health information technology for economic and clinical health act. 2009

[4] Buntin MB, Burke MF, Hoaglin MC, Blumenthal D (2011). The benefits of health information technology: a review of the recent literature shows predominantly positive results. Health Aff (Millwood).

[5] Calisir F, Calisir F (2004). The relation of interface usability characteristics, perceived usefulness, and perceived ease of use to end-user satisfaction with enterprise resource planning (ERP) systems. Comput Hum Behav.

[6] Tao D, Or CK (2013). Effects of self-management health information technology on glycaemic control for patients with diabetes: a meta-analysis of randomized controlled trials. J Telemed Telecare.

[7] Singh K, Drouin K, Newmark LP, Rozenblum R, Lee J, Landman A, Pabo E, Klinger EV, Bates DW (2016). Developing a framework for evaluating the patient engagement, quality, and safety of mobile health applications. Issue Brief (Commonw Fund).

[8] Baxter SK, Blank L, Woods HB, Payne N, Rimmer M, Goyder E (2014). Using logic model methods in systematic review synthesis: describing complex pathways in referral management interventions. BMC Med Res Methodol.

[9] Sutcliffe K, Thomas J, Stokes G, Hinds K, Bangpan M (2015). Intervention component analysis (ICA): a pragmatic approach for identifying the critical features of complex interventions. Syst Rev.

[10] Lewis TL, Wyatt JC (2014). mHealth and mobile medical apps: a framework to assess risk and promote safer use. J Med Internet Res.

[11] Collins LM, Murphy SA, Strecher V (2007). The multiphase optimization strategy (MOST) and the sequential multiple assignment randomized trial (SMART): new methods for more potent eHealth interventions. Am J Prev Med.

[12] Shibata N, Kajikawa Y, Sakata I (2010). Extracting the commercialization gap between science and technology—case study of a solar cell. Technol Forecast Soc Change.

[13] Yen P-Y, Bakken S (2012). Review of health information technology usability study methodologies. J Am Med Inform Assoc.

[14] Powell BJ, McMillen JC, Proctor EK, Carpenter CR, Griffey RT, Bunger AC, Glass JE, York JL (2012). A compilation of strategies for implementing clinical innovations in health and mental health. Med Care Res Rev.

[15] Powell BJ, Waltz TJ, Chinman MJ, Damschroder LJ, Smith JL, Matthieu MM, Proctor EK, Kirchner JE (2015). A refined compilation of implementation strategies: results from the expert recommendations for implementing change (ERIC) project. Implement Sci.

[16] Bickman L (2008). A measurement feedback system (MFS) is necessary to improve mental health outcomes. J Am Acad Child Adolesc Psychiatry.

[17] Bickman L, Lyon AR, Wolpert M (2016). Achieving precision mental health through effective assessment, monitoring, and feedback processes. Adm Policy Ment Health.

[18] Roe D, Drake RE, Slade M (2015). Routine outcome monitoring: an international endeavour. Int Rev Psychiatry.

[19] Bickman L, Kelley SD, Breda C, de Andrade AR, Riemer M (2011). Effects of routine feedback to clinicians on mental health outcomes of youths: results of a randomized trial. Psychiatr Serv.

[20] Lambert MJ, Whipple JL, Hawkins EJ, Vermeersch DA, Nielsen SL, Smart DW (2003). Is it time for clinicians to routinely track patient outcome? A meta-analysis. Clin Psychol Sci Pract.

[21] Results from the 2013 National Survey on Drug Use and Health: Summary of National Findings. Rockville: Substance Abuse and Mental Health Services Administration; 2013. http://www.samhsa.gov/data/sites/default/files/NSDUHresultsPDFWHTML2013/Web/NSDUHresults2013.pdf. 27656739

[22] Scott K, Lewis CC (2015). Using measurement-based care to enhance any treatment. Cogn Behav Pract.

[23] Kellermann AL, Jones SS (2013). What it will take to achieve the as-yet-unfulfilled promises of health information technology. Health Aff (Millwood).

[24] Bergen M, Peteraf MA (2002). Competitor identification and competitor analysis: a broad-based managerial approach. Manag Decis Econ.

[25] Blount A, Schoenbaum M, Kathol R, Rollman BL, Thomas M, O’Donohue W, Peek CJ (2007). The economics of behavioral health services in medical settings: a summary of the evidence. Prof Psychol Res Pract.

[26] Kazdin AE (2007). Mediators and mechanisms of change in psychotherapy research. Annu Rev Clin Psychol.

[27] Lewis CC, Boyd M, Beidas RS, Lyon AR, Chambers D, Aarons GA, Mittman B (2015). A research agenda for mechanistic dissemination and implementation.

[28] Waltz TJ, Powell BJ, Matthieu MM, Damschroder LJ, Chinman MJ, Smith JL, Proctor EK, Kirchner JE (2015). Use of concept mapping to characterize relationships among implementation strategies and assess their feasibility and importance: results from the expert recommendations for implementing change (ERIC) study. Implement Sci.

[29] Proctor EK, Powell BJ, McMillen JC (2013). Implementation strategies: recommendations for specifying and reporting. Implement Sci.

[30] Aarons GA, Hurlburt M, Horwitz SM (2010). Advancing a conceptual model of evidence-based practice implementation in public service sectors. Adm Policy Ment Health Ment Health Serv Res.

[31] Damschroder LJ, Aron DC, Keith RE, Kirsh SR, Alexander JA, Lowery JC (2009). Fostering implementation of health services research findings into practice: a consolidated framework for advancing implementation science. Implement Sci.

[32] Insel T, Cuthbert B, Garvey M, Heinssen R, Pine DS, Quinn K, Sanislow C, Wang P (2010). Research domain criteria (rdoc): toward a new classification framework for research on mental disorders. Am J Psychiatry.

[33] Hsu C-C, Sandford BA (2007). The Delphi technique: making sense of consensus. Pract Assess Res Eval.

[34] Howe GW, Beach SRH, Brody GH (2010). Microtrial methods for translating gene-environment dynamics into preventive interventions. Prev Sci.

[35] Leijten P, Dishion TJ, Thomaes S, Raaijmakers MAJ, Orobio de Castro B, Matthys W (2015). Bringing parenting interventions back to the future: how randomized microtrials may benefit parenting intervention efficacy. Clin Psychol Sci Pract.

[36] Lyon AR, Koerner K (2016). User-centered design for psychosocial intervention development and implementation. Clin Psychol Sci Pract.

[37] Kluger AN, DeNisi A (1996). The effects of feedback interventions on performance: a historical review, a meta-analysis, and a preliminary feedback intervention theory. Psychol Bull.

[38] Riemer M, Rosof-Williams J, Bickman L (2005). Theories related to changing clinician practice. Child Adolesc Psychiatr Clin N Am.

[39] Courage C, Baxter K. Understanding your users: a practical guide to user requirements: methods, tools, and techniques. San Francisco, CA :Gulf Professional Publishing; 2005.

[40] Pea RD (1987). User centered system design: new perspectives on human-computer interaction. J Educ Comput Res.

[41] Eden J, Maslow K, Le M, Blazer D. et al. The mental health and substance use workforce for older adults: in whose hands? San Francisco, CA: National Academies Press; 2012 24851291

[42] DIS I: 9241–210: 2010. Ergonomics of human system interaction-Part 210: human-centred design for interactive systems. Int Stand Organ ISO Switz 2009.

[43] Rubin J, Chisnell D (2008). Handbook of usability testing: how to plan, design and conduct effective tests.

[44] Proctor E, Silmere H, Raghavan R, Hovmand P, Aarons G, Bunger A, Griffey R, Hensley M (2010). Outcomes for implementation research: conceptual distinctions, measurement challenges, and research agenda. Adm Policy Ment Health Ment Health Serv Res.

[45] Rogers EM (2010). Diffusion of innovations.

[46] Tabak RG, Khoong EC, Chambers DA, Brownson RC (2012). Bridging research and practice: models for dissemination and implementation research. Am J Prev Med.

[47] Douglas S, Button S, Casey SE (2014). Implementing for sustainability: promoting use of a measurement feedback system for innovation and quality improvement. Adm Policy Ment Health Ment Health Serv Res.

[48] Edbrooke-Childs J, Wolpert M, Deighton J (2016). Introduction to the special section on implementing feedback from outcome measures in child and adult mental health services. Adm Policy Ment Health Ment Health Serv Res.

[49] Lyon AR, Lewis CC (2015). Designing health information technologies for uptake: development and implementation of measurement feedback systems in mental health service delivery. Adm Policy Ment Health Ment Health Serv Res.

[50] Landes SJ, Carlson EB, Ruzek JI, Wang D, Hugo E, DeGaetano N, Chambers JG, Lindley SE (2015). Provider-driven development of a measurement feedback system to enhance measurement-based care in VA mental health. Cogn Behav Pract.

[51] Lyon AR, Wasse JK, Ludwig K, Zachry M, Bruns EJ, Unützer J, McCauley E. The contextualized technology adaptation process (CTAP): Optimizing health information technology to improve mental health systems. Administration and Policy in Mental Health and Mental Health Services Research. 2016;43:394-409. 10.1007/s10488-015-0637-x PMC4536193 25677251

[52] Lyon AR, Lewis CC, Boyd MR, Hendrix E, Liu F (2016). Capabilities and characteristics of digital measurement feedback systems: results from a comprehensive review. Adm Policy Ment Health Ment Health Serv Res.

[53] Fernández-Alemán JL, Señor IC, Lozoya PÁO, Toval A (2013). Security and privacy in electronic health records: a systematic literature review. J Biomed Inform.

[54] Grant RW, Wald JS, Poon EG, Schnipper JL, Gandhi TK, Volk LA, Middleton B (2006). Design and implementation of a web-based patient portal linked to an ambulatory care electronic health record: Patient gateway for diabetes collaborative care. Diabetes Technol Ther.

[55] Staroselsky M, Volk LA, Tsurikova R, Pizziferri L, Lippincott M, Wald J, Bates DW (2006). Improving electronic health record (EHR) accuracy and increasing compliance with health maintenance clinical guidelines through patient access and input. Int J Med Inf.

[56] Hill CE, Thompson BJ, Williams EN (1997). A guide to conducting consensual qualitative research. Couns Psychol.

[57] Hill CE, Knox S, Thompson BJ, Williams EN, Hess SA, Ladany N (2005). Consensual qualitative research: an update. J Couns Psychol.

[58] Tang H, Ng JHK (2006). Googling for a diagnosis—use of Google as a diagnostic aid: internet based study. BMJ.

[59] Brin S, Page L (2012). Reprint of: the anatomy of a large-scale hypertextual web search engine. Comput Netw.

[60] Why do Google results vary in different locations? - LCN.com. Grow a successful website with the LCN.com Business Hub. 2014.

